# Caveolin-3 Promotes a Vascular Smooth Muscle Contractile Phenotype

**DOI:** 10.3389/fcvm.2015.00027

**Published:** 2015-06-11

**Authors:** Jorge L. Gutierrez-Pajares, Jeannette Iturrieta, Vipin Dulam, Yu Wang, Stephanos Pavlides, Gabriella Malacari, Michael P. Lisanti, Philippe G. Frank

**Affiliations:** ^1^Faculté de Médecine, INSERM UMR1069 “Nutrition, Croissance et Cancer”, Université François Rabelais de Tours, Tours, France; ^2^Department of Stem Cell Biology and Regenerative Medicine, Kimmel Cancer Center, Thomas Jefferson University, Philadelphia, PA, USA; ^3^Department of Cancer Biology, Kimmel Cancer Center, Thomas Jefferson University, Philadelphia, PA, USA; ^4^The Manchester Centre for Cellular Metabolism (MCCM), Institute of Cancer Sciences, University of Manchester, Manchester, UK; ^5^The Breakthrough Breast Cancer Research Unit, Institute of Cancer Sciences, University of Manchester, Manchester, UK; ^6^Department of Biochemistry and Molecular Biology, Thomas Jefferson University, Philadelphia, PA, USA

**Keywords:** caveolae, caveolin-3, smooth muscle, vascular diseases, lipoproteins

## Abstract

Epidemiological studies have demonstrated the importance of cardiovascular diseases in Western countries. Among the cell types associated with a dysfunctional vasculature, smooth muscle (SM) cells are believed to play an essential role in the development of these illnesses. Vascular SM cells are key regulators of the vascular tone and also have an important function in the development of atherosclerosis and restenosis. While in the normal vasculature, contractile SM cells are predominant, in atherosclerotic vascular lesions, synthetic cells migrate toward the neointima, proliferate, and synthetize extracellular matrix proteins. In the present study, we have examined the role of caveolin-3 in the regulation of SM cell phenotype. Caveolin-3 is expressed *in vivo* in normal arterial SM cells, but its expression appears to be lost in cultured SM cells. Our data show that caveolin-3 expression in the A7r5 SM cell line is associated with increased expression of contractility markers such as SM α-actin, SM myosin heavy chain but decreased expression of the synthetic phenotype markers such as p-Elk and Klf4. Moreover, we also show that caveolin-3 expression can reduce proliferation upon treatment with LDL or PDGF. Finally, we show that caveolin-3-expressing SM cells are less sensitive to apoptosis than control cells upon treatment with oxidized LDL. Taken together, our data suggest that caveolin-3 can regulate the phenotypic switch between contractile and synthetic SM cells. A better understanding of the factors regulating caveolin-3 expression and function in this cell type will permit the development of a better comprehension of the factors regulating SM function in atherosclerosis and restenosis.

## Introduction

The development of atherosclerosis is characterized by vascular wall accumulation of lipids, which can attract monocyte-derived macrophages followed by vascular smooth muscle cells (vSMC). Migrating and proliferating (i.e., intimal) SM cells have specific properties not normally observed in medial SM cells. These newly acquired properties include the ability of these cells to produce extracellular matrix proteins and a reduction in the expression of proteins involved in muscle contraction. These cells have been linked to a synthetic phenotype whereas medial SM cells have been associated with a contractile phenotype. Contractile cells are characterized by the expression of contractile proteins (such as calponin and SMα-actin). By contrast, synthetic cells are described with a reduced expression of contractile proteins and an increased expression of proteins involved in migration/invasion and proliferation as well as extracellular matrix protein production. Therefore, a synthetic phenotype is normally observed during embryonic differentiation of vessels while in the adult healthy vasculature, vSMCs normally present a contractile phenotype that regulates blood pressure and flow (1). However, during the development of diseases such as atherosclerosis and restenosis (2–4), the migration of vSMC from the media to the intima in response to the inflammatory milieu induces a switch from a contractile to a synthetic phenotype. One of the main transcription factors that regulates the vSMC phenotype is serum response factor (SRF) (5). SRF activity is finely regulated by co-transcription factors Elk-1 and myocardin (6, 7). For example, previous studies have demonstrated that phosphorylated Elk-1 (p-Elk-1) can repress the transcription of vSMC-specific genes such as SM 22α (7). By contrast, the myocardin/SRF complex upregulates key proteins that favor contractility: they include SM 22α, SM myosin heavy chain, and SM α-actin (8–11). In addition, Krüppel-like transcription factor 4 (Klf-4), a known repressor of vSMC contractile marker genes, has also been shown to induce a phenotypic switch toward the vSMC synthetic state (8, 12).

Caveolae are flask-shaped structures present at the plasma membrane. Caveolins were identified as essential proteins to form this invagination. Currently, three protein members of this family have been identified, and they are named caveolin-1, -2, and -3. Both caveolin-1 and -3 can induce caveolae formation while caveolin-2 requires caveolin-1 to reach the plasma membrane (13). Among them, caveolin-3 expression is restricted to muscle cells (14). In particular, vSMCs express all of the caveolin isoforms, which can interact with each other to form homo- or hetero-oligomers (15). In addition, studies in *Cav-1-* and *Cav-3*-deficient mice have indicated that caveolin-1 is responsible for the formation of the majority of caveolae in SMCs (15), suggesting that caveolin-3 may have an unidentified role in this cell type. While caveolin-1 has been implicated in the regulation of important signaling pathways, a similar role for caveolin-3 has been proposed. Caveolin-3 is expressed in cardiac, skeletal, and SM cells (14, 16). However, whereas fully differentiated cardiac and skeletal muscle cells express only the caveolin-3 isoform, vascular SM cells co-express either caveolin-1 and -2 (in veins) or caveolin-1, -2, and -3 (in arteries) (17). This observation suggests that, in arteries, caveolin-1 and -3 are co-expressed or that there are different sub-populations of SM cells, which express different caveolin proteins. Such heterogeneity of arterial smooth cells has previously been reported with other protein markers (18). It also appears that primary cultures of late passage arterial SMCs express only caveolin-1 and -2 but lack caveolin-3 (19). Interestingly, the proliferating primary cultured vSMCs present a phenotype (synthetic phenotype, as opposed to the contractile phenotype found in normal artery) that is similar to that of proliferating vSMCs found in atherosclerotic lesions (20). These observations may suggest that a change in the expression of caveolins occurs in atherosclerotic lesions and in cell cultures.

To better understand the role of caveolin-3 in the regulation of SM cell differentiation, we have examined its role in the regulation of SM cell proliferation, migration, and contractile properties. Our data suggest that caveolin-3 directly regulates a switch between proliferative and contractile vSMCs, and that regulating its expression may also lead to a better understanding of the factors regulating vascular function.

## Materials and Methods

### Reagents

Matrigel invasion chambers were obtained from BD Biosciences (Bedford, MA, USA). Recombinant human PDGF-BB (220-BB) was obtained from R&D Systems, Inc. (Minneapolis, MN, USA). Human LDL (BT-903) and oxidized LDL (oxLDL) (BT-910) were purchased from Biomedical Technologies, Inc. (Stoughton, MA, USA). Antibodies against SM 22α (ab73978), caveolin-3 (ab173575), SM α-actin (ab124964), and myocardin (ab22621) were obtained from Abcam (Cambridge, MA, USA). Anti-pan-Akt (2920), anti-phospho S473 Akt1 (9271), and anti-phospho Erk1/2 (Thr202/Tyr204) (9101) were obtained from Cell Signaling, Inc. (Danvers, MA, USA). Anti-Klf4 (LS-B5641) was obtained from LifeSpan Biosciences, Inc. (Seattle, WA, USA). Antibodies against caveolin-1 (sc-894), calponin (sc-28545), SRF (sc-335), SM myosin heavy chain (sc-6956), Erk1/2 (sc-135900), and phosphorylated Elk-1 (sc-8406) were obtained from Santa Cruz Biotechnology, Inc. (Dallas, TX, USA). Antibody against β-actin (A5441) was obtained from Sigma-Aldrich Corp. (St. Louis, MO, USA). Secondary HRP-conjugated antibodies against mouse and rabbit antibodies were obtained from Thermo Fisher Scientific, Inc. (Rockford, IL, USA). LDH cytotoxicity assay kit (10008882) was purchased from Cayman Chemical Company (Ann Arbor, MI, USA). Secondary Alexa Fluor^®^ 488 goat anti-mouse IgG (A11001), Alexa Fluor^®^ 594 goat anti-rabbit IgG (A11012), Alexa Fluor^®^ 680-conjugated phalloidin (A22286), DAPI (D3571), ProLong^®^ Gold antifade reagent (P36930) and phenol-free, high glucose Dulbecco’s Modified Eagle Medium (DMEM) were obtained from Life Technologies (Grand Island, NY, USA). BrdU Elisa colorimetric kit was obtained from Roche Diagnostics (Indianapolis, IN, USA).

### Cell culture

A7r5 cells were obtained from the American Type Culture Collection. They were routinely cultured in complete media [phenol red-free DMEM supplemented with l-glutamine, sodium pyruvate, penicillin and streptomycin, and 10% fetal bovine serum (FBS)].

### Stable transfection of A7r5 cells

Cells were infected with retroviral particles containing wild-type rat caveolin-3 cDNA (14, 21) or empty vector. Two days post-infection, cells were selected in the presence of puromycin for a week. After selection, cells were cultured in complete media.

### Confocal immunofluorescence

A7r5 cells were seeded on coverslips and incubated in complete media. After 3 days in culture, cells were fixed in 2% paraformaldehyde, permeabilized with Triton X-100, blocked with 3% BSA in PBS, and incubated with primary antibody for 1 h. Fluorescent-conjugated secondary antibodies were applied to the coverslips for 30 min. When appropriate, cells were washed with PBS and stained with DAPI and Alexa Fluor^®^ 680-conjugated phalloidin prior to mounting using ProLong^®^ Gold antifade reagent. Immunofluorescence images were visualized and captured using a Zeiss LSM 510 Meta Confocal Laser Scanning Microscope.

### Proliferation assays

Two types of experiments were performed to evaluate proliferation. In the first one, cells were seeded in 96-well plates (3,000 cells per well) with complete media. After 1 day, media was replaced with DMEM containing 0.1% BSA for 24 h (serum deprivation media, SDM), and cells were incubated with SDM containing increasing concentrations of LDL for an additional 48 h. Cells were then subjected to proliferation measurements using a BrdU incorporation assay following the manufacturer’s instructions. In the second experiment, 300,000 cells were seeded in 60-mm dishes and treated with SDM. After 24 h, 100 μg/mL LDL in SDM was added for 48 h. Cells were then trypsinized and loaded in a hemocytometer to be counted.

### Cell death/apoptosis measurements

Three thousand cells were seeded in 96-well plates and grown in SDM for 24 h. Cells were then cultured with increasing concentrations of oxLDL for 48 h. LDH activity in the culture supernatant was determined according to the manufacturer’s instructions.

### Wound-healing assay

Cell cultures were grown to 100% confluence in six-well plates using complete media. Once homogeneous monolayers of cells were obtained, cells were cultured in media supplemented with 1% FBS for 24 h. In each well, a confluent monolayer of cells was artificially wounded with a micropipette tip. Detached cells were removed by washing with PBS, and experiments were continued by incubating cells with 1% FBS in DMEM (control) or 40 ng/mL PDGF-BB. Wound closure was monitored by comparing digital photographs of the same region of interest taken at 0- and 24-h time points. Pictures were analyzed by using the Image J program developed by Dr. Wayne Rasband (National Institutes of Health, Bethesda, MD, USA). Additionally, at the end of the experiment, paraformaldehyde-fixed cells were stained with 0.1% crystal violet and photographed.

### Matrigel invasion assay

Twenty-five thousand cells per well were seeded on top of the insert (upper chamber) placed over a 24-well matrigel invasion plates (8-μm pore size). The lower chamber was filled with media containing 0.1% BSA, 1% FBS, or 40 ng/mL PDGF-BB. After 16 h of culture, non-invading cells were removed from the side where cells had been seeded, and the side facing the lower chamber was stained with crystal violet. Pictures of four different fields under a 200× microscope magnification were taken to count the number of stained cells that invaded the matrigel chamber.

### Western blot

Cell protein extraction was conducted by incubating cells with a RIPA lysis buffer containing phosphatase and protease inhibitors. Protein concentrations were determined using the BCA protein assay (Thermo Scientific). Proteins were separated by SDS-PAGE and then transferred to a nitrocellulose membrane. Ponceau red staining was used to confirm complete blotting. The membrane was blocked with 5% non-fat milk or BSA in PBS, washed with PBS, and incubated with primary antibody diluted in 5% BSA in PBS-Tween overnight at 4°C. After washing with PBS, the blot was incubated with HRP-coupled secondary antibody. ECL (Pierce) was used to develop antibody location via chemiluminescence. Densitometric analysis was performed using the Image J program.

### Statistical analysis

All data were expressed as mean ± SD. Data were analyzed using the Kruskal–Wallis Non-parametric ANOVA test with Dunn’s Multiple Comparison post-test or the Mann–Whitney test. All data were processed using INSTAT v. 3.05 (GraphPad Software, La Jolla, CA, USA). Statistical significance was established at *P* < 0.05.

## Results

### Smooth muscle cell caveolin-3 expression is associated with a switch toward a contractile phenotype

**Figure 1:**
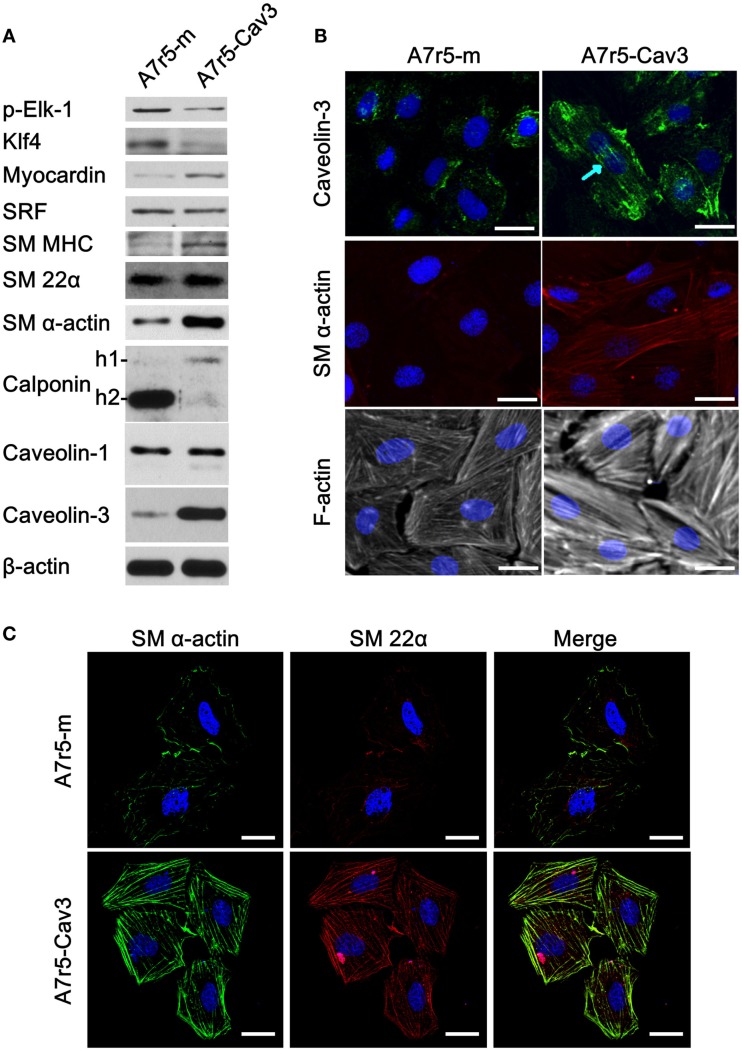
**Expression of caveolin-3 promotes the switch to a contractile phenotype of vSMCs**. A7r5 cells were stably transfected with caveolin-3 cDNA (A7r5-Cav3) or vector alone (A7r5-m) and selected with puromycin. Stably transfected cells were seeded and collected 48 h later (80% confluent). **(A)** Thirty micrograms of total protein was loaded onto polyacrylamide gels, transferred to nitrocellulose, and incubated with antibodies against transcription factors relevant for the control of a contractile phenotype [p-Elk-1, Myocardin, and Krüppel-like Factor 4 (Klf4)] and protein markers of a contractile phenotype [smooth muscle Myosin Heavy Chain (SM MHC), smooth muscle 22α (SM 22α), smooth muscle α-actin (SM α-actin), and calponin (-1 and -2)]. Caveolin-3 protein detection was performed to confirm its expression and β-actin protein detection was used as a loading control. **(B)** Caveolin-3, SM α-actin, and F-actin were localized by immunofluorescence confocal microscopy. DAPI staining was used to detect the nucleus. The blue arrow points to stress fiber-like structures. Scale bar represents 25 μm. **(C)** Co-localization of SM α-actin and SM 22α was examined by confocal microscopy. DAPI staining was used to detect the nucleus. Scale bar represents 25 μm.

Since caveolin-3 protein is lost in cultured cells, we decided to express caveolin-3 in A7r5 cells using the pBABE retrovirus expression vector (Figure 1). Expression of caveolin-1 as well as other markers of SM differentiation was examined. We found that caveolin-3 expression in A7r5 cells (A7r5-Cav3) was associated with a robust increase in the expression levels of contractile protein markers (myocardin, SM myosin heavy chain, SM α-actin) and a reduction in the levels of protein markers of the synthetic phenotype (p-Elk-1, KLF4) compared to control-transduced cells (A7r5-m) (Figure 1A). As expected, SRF showed no difference in protein expression levels. Interestingly, we observed a clear switch in the calponin isoforms expressed in these cells. According to Uniprot (Uniprot, http://www.uniprot.org, last accessed on February 20, 2015), rat h1-calponin (or SM calponin-H1, Accession # Q08290) and h2-calponin (Accession # D3ZRX9) have a predicted molecular weight of 33 and 24 kDa, respectively. As demonstrated in Figure 1A, caveolin-3 expression drastically downregulated calponin-h2 while calponin-h1 was upregulated. No change in caveolin-1 or SM 22α expression was detected. Also, while A7r5 cells presented an ovoid-like shape, A7r5-Cav3 cells were characterized by a spindle-shaped cellular structure that has been associated with a contractile morphology.

To further characterize the effect of caveolin-3 on vSMC, immunofluorescence was conducted to detect caveolin-3, SM α-actin, and F-actin (Figure 1B). Caveolin-3 not only associated with the plasma membrane but also appeared to distribute along stress fiber-like structures. Noticeably, caveolin-3 expression also caused SM α-actin to form a well-defined actin network, and F-actin staining was more pronounced. Conversely, absence of caveolin-3 was associated with a less bundled and less organized actin cytoskeleton. Additionally, in another set of experiments, the intracellular localization of SM α-actin and SM 22α was compared. In Figure 1C, both proteins were found to co-localize in well-defined structures only when caveolin-3 was overexpressed. These structures resemble that of an organized contractile machinery (22).

### Caveolin-3 inhibits vSMC proliferation stimulated by LDL

**Figure 2:**
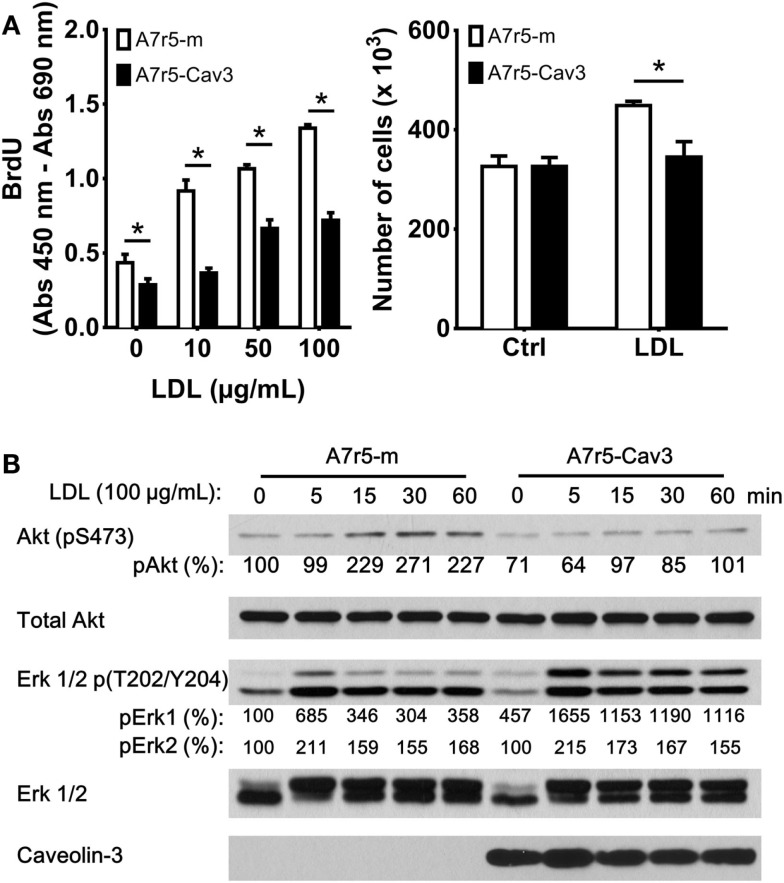
**A7r5-Cav3 cells are resistant to LDL-induced proliferation**. **(A)** Cells were seeded in complete culture media that was replaced with SDM 24 h later. *Left panel*. Cells in 96-well plates were incubated with 0, 10, 50, or 100 μg/mL LDL for an additional period of 48 h. BrdU incorporation was then performed, and the corresponding ELISA assay was realized according to the manufacturer’s instructions. *Right panel*. A7r5 cells (3 × 10^5^ cells in 6-cm dishes) were incubated with 100 μg/mL LDL for 48 h, and cells were then trypsinized and counted. *P < 0.05. **(B)** Cells were seeded in 6-cm dishes in complete media, cultured in SDM for 24 h, and then exposed to 100 μg/mL LDL for 5, 15, 30, or 60 min. After the corresponding exposure time, cells were collected and lysed. Thirty micrograms of total protein was loaded onto polyacrylamide gels, transferred to nitrocellulose, and incubated with antibodies against phosphorylated Akt1, total Akt, phosphorylated Erk 1/2, total Erk 1/2, or caveolin-3. Primary antibodies were detected with HRP-conjugated secondary antibodies. Relative levels of phosphorylated Akt, Erk1/2 were quantified by densitometry and expressed as the percentage of non-LDL treated A7r5-m cells (control group).

Earlier studies have indicated that SM phenotype influences the ability of this cell type to bind and internalize LDL (23). We therefore decided to analyze the effect of caveolin-3 in the regulation of cellular proliferation in the presence of LDL, which has also been shown to promote vSMC proliferation (24, 25) (Figure 2A). Initially, the replicating cell index was determined by BrdU incorporation. This approach showed that exposure of A7r5-m cells to 10, 50, and 100 μg/mL LDL significantly accelerated the progression of vSMC to the S phase compared to A7r5-Cav3 cells. Also, incubation of cells with 100 μg/mL LDL showed an increased number of viable A7r5-m cells compared to A7r5-Cav3 cells after 48 h of exposure to LDL (Figure 2A). Previous studies have shown that the Akt-signaling pathway can regulate vSMC proliferation (26). To better understand the mechanisms associated with caveolin-3 inhibition of LDL-induced cell proliferation, we evaluated the activation level of Akt1. As shown in Figure 2B, LDL-induced phosphorylation of Akt at serine 473 was reduced in the presence of caveolin-3, therefore indicating that Akt remained inactive in A7r5-Cav3 cells in our 60-min period of incubation with LDL (Figure 2B). On the contrary, activation of Erk1/2 reached a peak 5 min after LDL treatment of A7r5-m cells, while a sustained activation of Erk1/2 was observed in A7r5-Cav3 cells.

### Caveolin-3 reduces cell motility of vSMC

**Figure 3:**
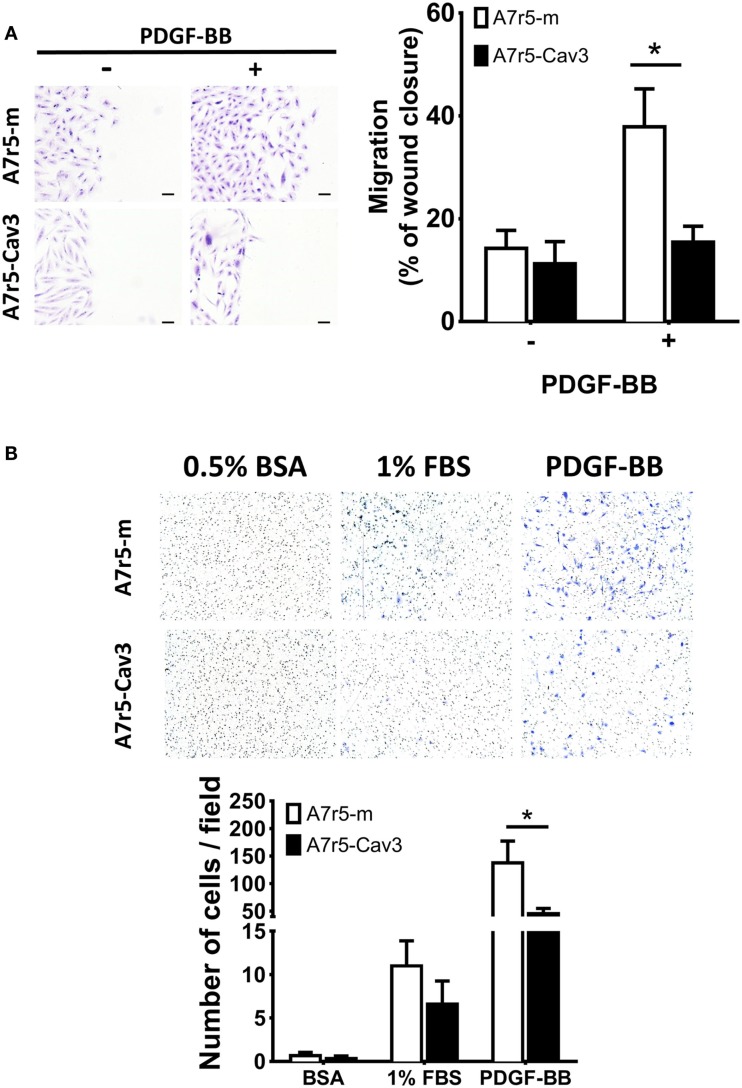
**A7r5-Cav3 cells display reduced migratory response to PDGF**. **(A)** 4 × 10^5^ cells were seeded in six-well plates; complete media was replaced with SDM for 24 h. A 100% confluent cell culture was submitted to a wound with a pipette tip and washed, and 50 ng/mL PDGF was added. After 8 h of culture, cells were washed, fixed, and stained with crystal violet. Pictures were taken at 0- and 8-h time points. *Left panel*. Representative pictures of stained fields are shown at the end of the experiment of cells treated (+) or not (−) with PDGF. Scale bar represents 0.1 mm. *Right panel*. Graph represents the percentage of area cell-free (% of wound closure) remaining after 8 h of PDGF exposure (+) or vehicle alone (−) for three independent experiments. *P < 0.05. **(B)** In an invasion chamber, 25 × 10^3^ cells were seeded over matrigel in the upper chamber with medium containing 0.1% BSA. Medium containing 0.5% BSA, 1% FBS, or 50 ng/mL PDGF was added to the lower chamber. After 16 h of culture, the upper chamber was removed, cleaned, and stained with crystal violet. *Upper panel* shows representative images of fields viewed under the microscope (200×). *Lower panel* shows the quantification of the number of cells per field. White and black bars represent A7r5-m and A7r5-Cav3 cells, respectively. *P < 0.05.

Another property associated with the SM synthetic phenotype is the ability of cells to migrate in response to a stimulating factor such as PDGF (27). In the following experiment, the 2-D migration response was first examined using a wound-healing assay. As observed in Figure 3A, A7r5-Cav3 cells hardly responded to an 8-h period of exposure to PDGF-BB compared to A7r5-m cells (*P* < 0.05). This observation was in sharp contrast with the control cells that were able to fill 40% of the wound after 8 h in the presence of PDGF. Importantly, no difference was observed under basal conditions (without stimulus) using cells expressing or not caveolin-3. Noticeably, while A7r5-m cells presented an ovoid-like shape, A7r5-Cav3 cells were characterized by a spindle shape that has been associated with a more differentiated contractile phenotype. To gain insight into the role of caveolin-3 in the invasive capacity of vSMC, A7r5 cells were seeded over a matrigel layer and PDGF-BB or FBS were used as chemoattractants (Figure 3B). We show that expression of caveolin-3 prevented the invasion of A7r5 cells when compared to control cells (*P* < 0.05). Similar levels of invasion for both types of cells were observed when exposed to BSA or FBS.

### Caveolin-3 prevents vSMC cell death

**Figure 4:**
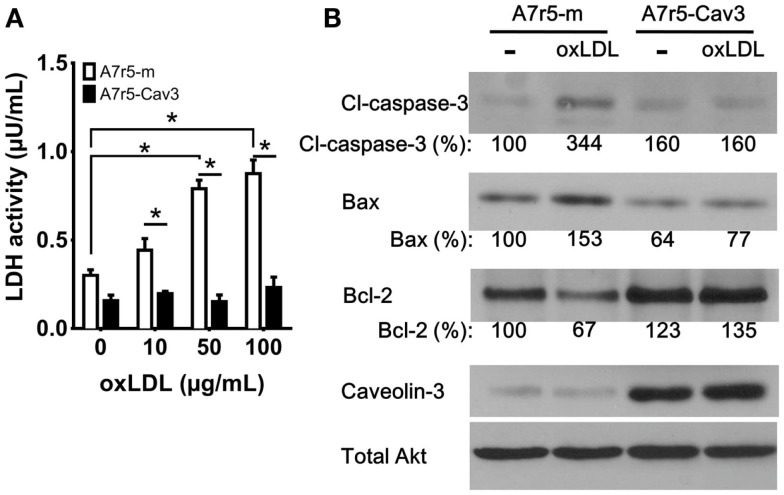
**Caveolin-3 prevents oxidized LDL-induced cell death**. Cells were seeded in complete media, and, 24 h later, media was replaced with SDM containing oxidized LDL (oxLDL). **(A)** 3 × 10^3^ cells were seeded in 96-well plates and exposed to increasing concentrations of oxLDL for 24 h. LDH activity in the culture media was determined according to the manufacturer’s instructions. A graph displaying the average of three experiments is shown. *P < 0.05. **(B)** Western blot analysis was performed to detect the levels of cleaved caspase-3 (Cl-caspase-3), Bax, Bcl-2, caveolin-3, and total Akt from cells treated with 50 μg/mL oxLDL for 24 h. Relative levels of Cl-caspase-3, Bax, and Bcl-2 were quantified by densitometry and expressed as the percentage of non-oxLDL treated A7r5-m cells (control group).

Previous studies have shown that the importance of vSMC apoptosis is directly related to plaque rupture (28, 29). To explore a potential protective role of caveolin-3, vSMC were exposed to oxLDL particles to induce cell death. As observed in Figure 4A, LDH activity in the culture media of A7r5-m cells showed a significant increase in all treated groups compared to A7r5-Cav3 cells. A dose-dependent curve was also observed only in the case of A7r5-m cells. It is inferred that caveolin-3 expression significantly precluded oxLDL-induced cell death. In support of this hypothesis, A7r5-m cells responded to oxLDL treatment by upregulating the pro-apoptotic proteins cleaved Caspase-3 and Bax and downregulating the anti-apoptotic protein Bcl-2. No changes of this type were detected in A7r5-Cav3 cells (Figure 4B). Interestingly, Bcl-2 expression in A7r5-Cav3 cells was even increased under basal conditions.

## Discussion

vSMCs typically regulate the contraction of blood vessels, and their function is finely regulated by signaling molecules sent by endothelial cells (30). The vSMC cell state is regulated by key co-transcription factors such as Klf4, Elk-1, and myocardin. These co-transcription factors bind to SRF to regulate the transcription of genes encoding proteins involved in the contraction machinery (6, 7). Klf4 is upregulated in response to vascular injury (synthetic phenotype) and represses the expression of vSMC genes (10). Active Elk-1 (phosphorylated Elk-1) has been shown to regulate the expression of c-fos during stimulated DNA synthesis in vSMC (31) and displace myocardin from SRF by motif competition, enhancing repression of vSMC genes (6, 32). On the other hand, myocardin has been shown to coordinate the expression of genes that promote the vSMC contractile phenotype (6, 11). It can be inferred that the vascular SM cell type is not terminally differentiated and can switch between the two phenotypes. This idea is supported by the fact that cells obtained from freshly prepared cultures of adult aortic vSMC turn on genes associated with the synthetic phenotype while those associated with the contractile one are turned off (33). In the present work, caveolin-3-mediated changes in the expression of these key co-transcription factors were identified. It was observed that expression of caveolin-3 caused a downregulation of active Elk-1 and Klf4 and an upregulation of myocardin, while no changes in SRF were detected (Figure 1). Furthermore, caveolin-3 was able to alter the synthetic phenotype and promote the acquisition of contractile properties. In addition, expression of caveolin-3 led to a diminished response to proliferative and chemoattractant stimulus. This effect of caveolin-3 is in line with the observation that caveolin-3, but not caveolin-1, was downregulated when the contractile-to-synthetic transition occurred in vSMC (19). These findings are also consistent with the previously demonstrated role of caveolin-3 in skeletal myoblast differentiation (14).

### Caveolin-3 and the regulation of a contractile phenotype

In agreement with the upregulation of myocardin observed in caveolin-3-expressing cells, an upregulation of contractile protein markers (SM MHC and SM α-actin) was detected. Importantly, a clear switch in calponin isoforms expression was detected where the h2-calponin and h1-calponin were down and upregulated in caveolin-3-expressing vSMCs, respectively. The observed reduction in h2-calponin may be associated with its role in inhibiting cellular proliferation while h1-calponin is expressed in quiescent differentiated vSMCs (34). These molecular changes observed with caveolin-3 expression were also evident by immunofluorescence analysis where SM α-actin expression was increased and a cytoskeletal reorganization accompanied the formation of a more clearly defined F-actin network associated with a contractile phenotype.

Moreover, even if caveolin-3 did not affect SM 22α protein levels (Figure 1A), caveolin-3 induced a clear reorganization of the distribution of SM 22α, which associated with the contractile machinery and co-localized with SM α-actin (Figure 1C). The upregulation of SM α-actin and its observed intracellular distribution pattern suggest an enhanced contractile activity, as previously described in other cell types (22). Interestingly, caveolin-3 was associated with the plasma membrane and stress fiber-like structures within the cells. This finding suggests that caveolin-3 may have two distinct functions in vSMC. Caveolin-3 may regulate signaling pathways at the plasma membrane levels but may also associate with the cytosketelal network, thereby directly regulating vSMC contraction.

Previous studies have shown that myocardin expression is regulated by intracellular calcium signaling in vSMCs (35). Since caveolin-3 plays an important role in the regulation of calcium metabolism (36), our data suggest that caveolin-3 may regulate calcium metabolism to promote myocardin upregulation and, as a consequence, increases the expression levels of contractile markers (37). Additionally, miR-145 and miR-143 transcription has been shown to be regulated by myocardin, and these microRNAs have also been demonstrated to play key roles in the regulation of SM cell phenotype (38). Importantly, reduction of KLF4 activity has been observed upon induction of miR-145 and miR-143. Therefore, the present study suggests that caveolin-3 may be a key regulation of vSMC function.

### Caveolin-3 and the regulation of a synthetic phenotype

In accordance with the quiescent contractile phenotype promoted by caveolin-3, a diminished cellular proliferation response was observed upon LDL treatment (Figures 2A,B). Previous studies have shown that LDL activates proliferative signals via the ROS/Erk-signaling pathway in vSMCs (24). In the present study, we also observed that LDL-induced activation of Akt1 was precluded by caveolin-3 expression. vSMC Akt1 signaling is essential for proliferation (39) and represses myocardin promoter activity (40). In that case, inhibition of the Akt1 signaling pathway by caveolin-3 may therefore be associated with increased myocardin activity. Remarkably, a sustained activation of Erk1/2 was observed in cells overexpressing caveolin-3 upon LDL stimulation. This finding is in agreement with previous studies showing that constitutively active Erk1/2 may be related to an antiproliferative effect and a potential role in the regulation of cellular differentiation (41–43). Yang et al. (40) have postulated that Spry1 and Spry4 can modulate FGFR and EGFR signaling in vSMC: while Spry4 inhibits both Erk1/2 and Akt pathway activation, Spry1 enhances Akt activation. Importantly, Cabrita et al. (44) have reported that Spry1 and Spry4 can physically interact with the C-terminus of caveolin-1 and that Spry1 and caveolin-1 cooperatively inhibit FGF2-mediated signaling. In the current study, LDL was used to induce cellular proliferation, and we expected both Akt and Erk1/2 signaling pathways to be inhibited by caveolin-3. However, caveolin-3 expression only inhibited the Akt pathway while a sustained activation of the Erk1/2 pathway was observed. Based on the aforementioned studies, caveolin-3 may bind/inhibit Spry1 and Spry4, causing inhibition of the Akt-signaling pathway, thereby allowing a prolonged activation of Erk1/2 mediated by LDL. Alternatively, caveolin-1 and caveolin-3 may be associated with distinct caveolae and bind different proteins, which may allow for a differential regulation of the Erk1/2 and Akt-signaling pathways. The opposite effects of caveolin-3 on Erk1/2 and Akt activation suggest that caveolin-3 may be an important modulator of signaling pathways triggered by LDL.

Cellular migration is another relevant feature of the vSMC synthetic phenotype. Previous studies have shown that PDGF induces migration of vSMC (27). Our study clearly indicates that this pathway is remarkably inhibited in caveolin-3-expressing cells (Figure 3). Moreover, PDGF-stimulated matrigel invasion was also inhibited by caveolin-3. Inhibition of Akt1 phosphorylation by caveolin-3 (Figure 2B) could also explain the low migratory response since this kinase has been shown to play an important role in the regulation of cell migration (39).

### Regulation of caveolin-3 transcription and expression

In cultured vSMC, caveolin-3 is not regulated at the post-translation level but most likely at the transcriptional level. In support of this hypothesis, we have not observed any change in caveolin-3 protein expression after 20 passages in our model system. Our current findings indicate that transcription factors such as SRF, regulated by myocardin, may be essential for the transcription of the *CAV-3* gene, and that their downregulation or inhibition upon phenotyping switch may lead to reduced transcription of the *CAV-3* gene. Little is known about the mechanism regulating transcription of the caveolin-3 gene in vSMCs. However, in skeletal muscles, previous studies have shown that transcription of the *CAV-3* gene is enhanced by transcription factors such as TEAD4 (45), myogenin (46), or RORα (47). Interestingly, recent studies by Martínez-Moren et al. (48) have shown that exposure of C2C12 myoblasts to nitric oxide (NO) is associated with reduced transcription of the *Cav-3* gene. In that case, it was shown that S-nitrosylation of the muscular transcription factor myogenin led to a reduced myogenin transcriptional activity, which was responsible for the decrease in caveolin-3 mRNA levels. Transposed to the vasculature, this finding may suggest that NO production by endothelial cells could also directly affect vSMC caveolin-3 levels by inhibiting myocardin activity and therefore regulate vascular relaxation. Accordingly, reduced caveolin-3 protein levels may be associated with increased relaxation of the vasculature. Since expression of caveolin-3 in vSMC increases the levels of endogenous myocardin (Figure 1A), a reciprocal regulation of the two proteins may be critical to ensure a proper contractile phenotype.

### Role of caveolin-3 in the development of atherosclerosis

The present study provides new evidence suggesting that caveolin-3 may regulate arterial SM cell phenotype. Our data suggest that caveolin-3 may also be an important player in the regulation of SM cell function *in vivo* and that caveolin-3 may also play a key role in the development of atherosclerosis. Caveolin-3 may be one of the first proteins to be downregulated before migrating into the arterial intima, and its loss may, via its ability to regulate specific signaling cascades, accelerate the switch between contractile and synthetic phenotypes. Under these conditions, caveolin-3 may have an anti-atherogenic role and reduce migration and proliferation of vSMC. Signals that may regulate caveolin-3 protein levels in arterial vSMCs may include oxidized lipoproteins or cytokines. Loss of caveolin-3 may trigger a downregulation of myocardin, which has been shown to play a critical role in the regulation of essential properties associated with a contractile and non-inflammatory phenotype (49). Our data showing that caveolin-3 expression is reduced upon atheroma formation in *Apoe^−/−^* mouse aorta (Frank et al., unpublished results) are consistent with a role for caveolin-3 in the maintenance of vSMC contractile phenotype.

Intimal vSMCs exposed to oxLDL have also been shown to be susceptible to apoptosis (50, 51). As shown in Figure 4A, upon oxLDL exposure, A7r5-m cells underwent cell death by upregulating the pro-apoptotic proteins Bax and cleaved-caspase-3 and downregulating the anti-apoptotic protein Bcl-2 (Figure 4B). By contrast, caveolin-3 expression was associated with resistance to apoptosis in A7r5-Cav3 cells. The absence of effects of oxLDL in the presence of caveolin-3 may be due to the lack of efficient internalization of lipoproteins possibly due to reduced levels of scavenger receptors, such as CD36, OLR1 (aka, LOX-1), or SR-AI. This hypothesis is consistent with the observation that synthetic vSMC can form foam cells in a manner similar to macrophages (52). These data suggest that caveolin-3 expression may allow cells to maintain a contractile phenotype and reduce the deleterious effects of oxLDL. OxLDL can induce apoptosis by modulating Bax/Bcl-2 through its receptor OLR1 in vSMCs (28). Previous studies have shown that OLR1 is palmitoylated and uses the caveolae/raft-dependent endocytosis pathway to internalize its ligand (53, 54). Since we observed a minimal co-localization of caveolin-1 and caveolin-3 in A7r5-Cav3 cells (data not shown), OLR1 may be re-locating outside of caveolin-1-rich caveolae to caveolin-3-rich caveolae and may, as a consequence, inhibit downstream signaling pathways induced by oxLDL in A7r5-Cav3 cells.

Atherosclerosis is characterized by increased vSMC migration and proliferation in the intima during lesion formation. Therefore, a better understanding of the factors regulating caveolin-3 transcription may allow the development of new treatments that could control vSMC proliferation and migration, and therefore, atheroma formation. In addition, since vSMCs apoptosis and extracellular matrix protein synthesis play essential roles in the regulation of the vulnerable plaque formation (55), these treatments would also be applicable to prevent plaque rupture in patients at high risk for coronary artery diseases.

In conclusion, caveolin-3 promotes vSMC contractile phenotype and reduces cell proliferation and migration. Our findings demonstrate that caveolin-3 is a key protein that may be downregulated during atherosclerosis development or restenosis to promote vSMC dedifferentiation.

## Conflict of Interest Statement

The authors declare that the research was conducted in the absence of any commercial or financial relationships that could be construed as a potential conflict of interest.
